# Demographic factors associated with smoking cessation during pregnancy in New South Wales, Australia, 2000–2011

**DOI:** 10.1186/s12889-015-1725-2

**Published:** 2015-04-18

**Authors:** Erin Passmore, Rhydwyn McGuire, Patricia Correll, Jason Bentley

**Affiliations:** NSW Ministry of Health, North Sydney, NSW Australia; School of Public Health and Community Medicine, University of New South Wales, Sydney, NSW Australia

**Keywords:** Smoking, Smoking cessation, Smoking in pregnancy, Epidemiology, Australia

## Abstract

**Background:**

Smoking during pregnancy increases the risk of adverse health outcomes for both the mother and the child. Rates of smoking during pregnancy, and rates of smoking cessation during pregnancy, vary between demographic groups. This study describes demographic factors associated with smoking cessation during pregnancy in New South Wales, Australia, and describes trends in smoking cessation in demographic subgroups over the period 2000 – 2011.

**Methods:**

Data were obtained from the New South Wales Perinatal Data Collection, a population-based surveillance system covering all births in New South Wales. Multivariate logistic regression was used to explore associations between smoking cessation during pregnancy and demographic factors.

**Results:**

Between 2000 and 2011, rates of smoking cessation in pregnancy increased from 4.0% to 25.2%. Demographic characteristics associated with lower rates of smoking cessation during pregnancy included being a teenage mother, being an Aboriginal person, and having a higher number of previous pregnancies.

**Conclusions:**

Between 2000 and 2011, rates of smoking cessation during pregnancy increased dramatically across all demographic groups. However, specific demographic groups remain significantly less likely to quit smoking, suggesting a need for targeted efforts to promote smoking cessation in these groups.

## Background

Smoking during pregnancy increases the risk of adverse outcomes for mother and infant. For the mother it is a major risk factor for ectopic pregnancy, premature rupture of the membranes, placental abruption, placenta praevia, spontaneous abortion, stillbirth, coronary heart disease, stroke, peripheral vascular disease, cancer and a variety of other diseases and conditions [1-4]. For the infant, maternal smoking contributes to an increased risk of being born at an earlier gestational age, lower birth weight, being born with congenital conditions [1-6], sudden infant death syndrome, as well as poorer health in early childhood [7-11].

In Australia, rates of smoking during pregnancy have declined over the last decade, from 19.0% in 2001 [12] to 13.2% in 2011 [13]. However, rates of smoking during pregnancy vary between demographic groups. Rates of smoking in pregnancy are elevated among teenage mothers, Aboriginal and Torres Strait Islander women, women who live outside major cities, and women of low socioeconomic status [14,15]. Moreover, within New South Wales, although rates of smoking in pregnancy have declined in all demographic groups, the largest decreases have been among the most socioeconomically advantaged groups, with a relatively smaller decline among women of lower socioeconomic status, Aboriginal women and women living outside major cities, suggesting that inequalities in smoking in pregnancy have increased over time [14].

Relatively little data has been published on rates of smoking cessation during pregnancy in Australia. A Western Australian birth cohort study in 2002–03 found approximately 34% of women who smoked before pregnancy reported stopping smoking during pregnancy [16], while a New South Wales population-based study found that rates of smoking cessation during pregnancy declined from 4.5% in 1999 to 3.3% in 2003 [17]. However, like smoking during pregnancy, rates of smoking cessation during pregnancy also vary between demographic groups. Lu and colleagues reviewed nine cohort studies of smoking in pregnancy and found that the determinants of smoking cessation during pregnancy included maternal age, duration of smoking and number of cigarettes smoked per day, education level, socioeconomic status, and parity [18]. Similarly, a New South Wales study found that Aboriginal women, teenage mothers, women with previous children and women of lower socioeconomic status were less likely to stop smoking during pregnancy [17].

There is evidence for the effectiveness of interventions to support smoking cessation during pregnancy [19]. A recent systematic review concluded that smoking cessation interventions in pregnancy are effective in reducing the proportion of women who continue to smoke in late pregnancy, reducing the risk of low birth weight and preterm birth. There is also evidence that women consider provision of smoking cessation support during pregnancy to be appropriate [20-22]. However, success rates of smoking cessation interventions are often low, with interventions on average supporting an extra 6% of women to stop smoking during pregnancy, compared to usual care [19].

In order to develop successful maternal smoking cessation programs, the major determinants of smoking cessation during pregnancy need to be incorporated into intervention efforts, and interventions targeted towards women most at risk. Therefore, the aim of this study is to describe the demographic factors associated with smoking cessation during pregnancy in New South Wales, and to describe trends in smoking cessation in demographic subgroups over the period 2000 – 2011.

## Methods

### Study population and data source

Birth data were obtained from the New South Wales Perinatal Data Collection, a population-based surveillance system covering all live births and stillbirths of at least 20 weeks gestation or, when gestational age is unknown, at least 400 grams birth weight in New South Wales. Data collection occurs via a form completed by the attending midwife or doctor. The form includes information on maternal characteristics, pregnancy, labour and delivery factors and infant outcomes. The Perinatal Data Collection is a statutory data collection under the *New South Wales Public Health Act 2010*. De-identified unit record data was accessed and analysed by the authors in their role as employees of the New South Wales Ministry of Health. The study population for estimating overall smoking rates included all mothers residing in New South Wales who gave birth from 2000 to 2011, and for whom Aboriginality was recorded (n = 1,065,740). The study population for estimating smoking cessation was the subset of women who reported smoking in pregnancy (n = 147,961).

### Outcome measures

Smoking status during pregnancy was obtained from the Perinatal Data Collection. The Perinatal Data Collection does not have a specific indicator of smoking cessation during pregnancy, therefore smoking cessation needs to be derived from indicators of smoking in the first and second half of pregnancy. For the period 2000 to 2010, the Perinatal Data Collection form asked two questions about smoking during pregnancy: “Did the mother smoke at all during the pregnancy?” and “Number of cigarettes smoked each day on average in the second half of pregnancy”. Smoking cessation during pregnancy was defined as women who reported smoking during pregnancy, but reported smoking on average zero cigarettes per week in the second half of pregnancy. In 2011, the data collection form for the Perinatal Data Collection was revised to ask the following two questions about smoking behaviour: “Did the mother smoke at all during the first half of pregnancy?” and “Did the mother smoke at all during the second half of pregnancy?”. Therefore in 2011 smoking was defined as smoking in either the first or second half of pregnancy, and smoking cessation was defined as smoking during the first half of the pregnancy but not smoking in the second half of the pregnancy.

### Covariates

The following demographic characteristics were obtained from the Perinatal Data Collection: maternal age, whether antenatal care was received in the first 20 weeks, country of birth, self-reported Aboriginality of the mother, whether the birth was in a public or private hospital, parity, year of the birth, remoteness of residence and socioeconomic status. Remoteness was assigned based on the Accessibility/Remoteness Index of Australia (ARIA+) [23] according to the Statistical Local Area (SLA) of residence. The Australian Bureau of Statistics Socioeconomic Index for Areas (SEIFA) Index of Education and Occupation [24] was used to create socioeconomic quintiles based on SLA of residence. Both SEIFA and ARIA+ were assigned on the basis of the 2006 census.

### Analysis

Overall rates of smoking and smoking cessation were calculated for the whole study period in each of the above mentioned demographic groups. Multivariable logistic regression was carried out on the subset of women who reporting smoking during pregnancy to identify factors associated with smoking cessation. All analyses were performed using SAS 9.3.

## Results

### Smoking during pregnancy

**Figure 1:**
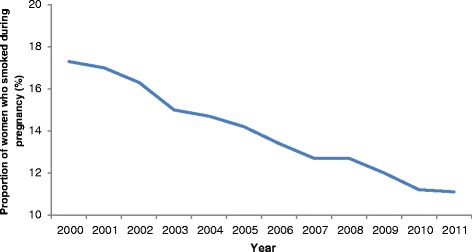
Proportion of women who smoked during pregnancy, New South Wales, 2000-2011.

Of the 1,065,740 women who gave birth in New South Wales between 2000 and 2011, 147,961 (13.9%) reported smoking during pregnancy. The proportion of women who smoked during pregnancy declined between 2000 and 2011, from 17.3% in 2000 to 11.1% in 2011 (Figure 1). The demographic characteristics of women who smoked during pregnancy are presented in Table 1. The highest rates of smoking were among Aboriginal women (53.4%), teenage mothers (38.9%), and women with three or more previous pregnancies (28.6%). The lowest rates of smoking in pregnancy were among private hospital patients (2.2%) and women in the most socioeconomically advantaged quintile (3.7%).

**Table 1:** **Rates of smoking and smoking cessation during pregnancy, New South Wales, 2000-2011**

		**Smoked at all during pregnancy**		**Ceased smoking during pregnancy**	
**Demographic characteristic**		**N**	%*	**N**	%**
**Hospital type**	Public	142,431	17.6	10,949	7.7
	Private	5,530	2.2	787	14.2
**SEIFA quintile (Education/Occupation)**	1st (most advantaged)	7,667	3.7	1,282	16.7
	2nd	19,884	9.4	1,856	9.3
	3rd	30,939	14.1	2,477	8.0
	4th	37,182	18.5	2,843	7.6
	5th (most disadvantaged)	52,246	22.9	3,277	6.3
**Country of birth and Aboriginality**	Australian born non-Aboriginal	114,007	15.8	8,501	7.5
	Aboriginal	16,198	53.4	593	3.7
	Non Australian born	17,756	5.7	2,642	14.9
**Maternal age (years)**	<20	15,841	38.9	937	5.9
	20-34	111,803	14.0	8,863	7.9
	35+	20,317	8.9	1,936	9.5
**Number of previous pregnancies**	0	51,250	11.5	6,777	13.2
	1	41,436	11.6	2,550	6.2
	2	27,076	16.8	1,361	5.0
	3+	28,080	28.6	1,044	3.7
**Remoteness (ARIA+)**	Major city	73,685	10.2	7,583	10.3
	Non major city	74,233	21.5	4,152	5.6
**Duration of pregnancy at first antenatal care visit**	Less than 20 weeks	117,672	12.4	9,979	8.5
	20 + weeks	26,912	25.1	1,528	5.7
**Total**		**147,961**	**13.9**	**11,736**	**7.9**

*Denominator: all women who gave birth **Denominator: women who smoked at all during pregnancy.

### Smoking cessation during pregnancy

**Figure 2:**
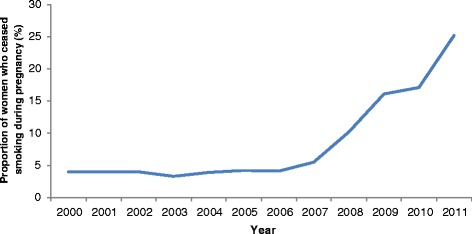
Proportion of women who ceased smoking during pregnancy*, New South Wales, 2000-2011. *Denominator: Women who smoked during pregnancy.

Of the 147,961 women who smoked during pregnancy in New South Wales between 2000 and 2011, 11,736 (7.9%) reported stopping smoking during pregnancy. The rate of smoking cessation during pregnancy was stable between 2000 and 2006, but increased markedly from 2007 onward (Figure 2). The demographic groups most likely to smoke during pregnancy were also the least likely to stop smoking during pregnancy. The poorest rates of smoking cessation during pregnancy were among Aboriginal women (3.7%) and women with three or more previous pregnancies (3.7%). The highest rates of smoking cessation in pregnancy were among women in the most socioeconomically advantaged quintile (16.7%), women born overseas (14.9%) and women who gave birth in private hospitals (14.2%).

In multivariable logistic regression analyses (Table 2), after adjusting for all other demographic characteristics, the strongest independent predictor of rates of smoking cessation in pregnancy was the year in which the baby was born, with the odds of smoking cessation increasing in each two year period between 2000–2001 and 2010–11. After adjusting for covariates, the odds of smoking cessation were higher among the following demographic groups: women who had their first antenatal care visit before 20 weeks gestation, women who gave birth in private hospitals, non-Aboriginal women, women with higher maternal age, women with fewer previous pregnancies, and women living in major cities.

**Table 2:** **Demographic factors associated with smoking cessation during pregnancy, New South Wales, 2001–2011**

**Demographic characteristic**		**Adjusted odds ratio**	**95% Confidence interval**	***P*** **-value**
**Year of birth**	2000-2001	Reference category		<0.001
	2002-2003	1.00	[0.91,1.09]	
	2004-2005	1.07	[0.93,1.24]	
	2006-2007	1.28	[1.05,1.57]	
	2008-2009	3.37	[2.51,4.52]	
	2010-2011	6.91	[5.50,8.69]	
**Duration of pregnancy at first antenatal visit**	<20 weeks	Reference category		<0.001
	20+ weeks	0.79	[0.73,0.85]	
**Hospital type**	Public	Reference category		<0.001
	Private	1.61	[1.29,2.01]	
**SEIFA quintile (Education/Occupation)**	1st (most advantaged)	1.70	[1.04,2.79]	
	2nd	1.07	[0.62,1.82]	
	3rd	1.34	[0.80,2.23]	
	4th	1.01	[0.61,1.68]	
	5th (most disadvantaged)	Reference category		0.116
**Country of birth and Aboriginality**	Australian born non-Aboriginal	Reference category		<0.001
	Aboriginal	0.55	[0.50,0.61]	
	Non Australian born	1.60	[1.44,1.78]	
**Maternal age (years)**	<20	0.53	[0.44,0.64]	
	20-34	0.87	[0.77,1.00]	
	35+	Reference category		<0.001
**Number of previous pregnancies**	0	Reference category		<0.001
	1	0.45	[0.41,0.49]	
	2	0.36	[0.32,0.39]	
	3+	0.27	[0.24,0.30]	
**Remoteness (ARIA+)**	Major city	Reference category		<0.001
	Non-major city	0.44	[0.31,0.62]	

### Demographic factors associated with smoking cessation during pregnancy – a comparison of 2000 and 2011

Comparing the two years 2000 and 2011, the rates of smoking cessation in pregnancy improved in all demographic groups (Table 3). However, most risk factors for continuing to smoke throughout pregnancy in 2000 remained risk factors in 2011. The notable exception was women who gave birth in public hospitals: in 2000, women who gave birth in public hospitals were less likely to stop smoking during pregnancy than women who gave birth in private hospitals, however in 2011 the reverse was true. The groups with the poorest rates of smoking cessation - Aboriginal women, and women with three or more previous pregnancies - remained the same in both time periods. The greatest absolute increase in cessation rates was in the two most advantaged socioeconomic quintiles and in women born overseas. The least improvement in cessation rates was among Aboriginal women, women giving birth in private hospitals, and teenage mothers.

**Table 3:** **Rates of smoking cessation during pregnancy by demographic factors, New South Wales, 2000 and 2011**

**Demographic characteristic**		**Rates of smoking cessation during pregnancy**				**Absolute increase in smoking cessation rate, 2000 to 2011**	**Multivariable logistic regression**			
		**2000**		**2011**			**2000**		**2011**	
		**No. of women who quit**	**% of smokers who quit**	**No. of women who quit**	**% of smokers who quit**		**Adjusted odds ratio**	**95% Confidence Interval**	**Adjusted odds ratio**	**95% Confidence Interval**
**Hospital type**	Public	530	3.8	2,607	25.2	21.4	Reference category		Reference category	
	Private	66	10.6	45	20.9	10.3	2.0	[1.1,3.6]	0.4	[0.2,0.5]
**SEIFA quintile (Education /Occupation)**	1st (most advantaged)	69	6.8	201	43.0	36.2	1.2	[0.5,2.7]	2.1	[1.6,2.8]
	2nd	145	6.0	413	33.3	27.3	1.2	[0.6,2.6]	1.6	[1.2,2.1]
	3rd	81	2.9	602	27.4	24.5	0.9	[0.5,1.5]	1.3	[1.0,1.6]
	4th	106	3.7	633	21.1	17.4	1.3	[0.8,2.0]	1.0	[0.8,1.3]
	5th (most disadvantaged)	195	3.5	803	22.1	18.6	Reference category		Reference category	
**Country of birth and Aboriginality**	Australian born non-Aboriginal	498	4.3	1,949	25.0	20.7	Reference category		Reference category	
	Aboriginal	16	1.4	212	13.7	12.3	0.5	[0.3,0.8]	0.6	[0.5,0.7]
	Non Australian born	82	4.3	491	40.3	36.0	1.1	[0.9,1.4]	1.6	[1.4,1.9]
**Maternal age (years)**	<20	82	5.0	197	19.8	14.8	1.1	[0.8,1.6]	0.7	[0.5,0.8]
	20-34	453	4.0	2,028	25.4	21.4	1.1	[0.8,1.4]	0.9	[0.8,1.1]
	35+	61	3.4	427	27.1	23.7	Reference category		Reference category	
**Number of previous pregnancies**	0	347	6.8	1,017	29.9	23.1	Reference category		Reference category	
	1	150	3.3	748	26.5	23.2	0.5	[0.4,0.6]	0.8	[0.7,0.9]
	2	55	2.1	480	23.8	21.7	0.3	[0.2,0.4]	0.7	[0.6,0.8]
	3+	44	1.8	407	17.8	16.0	0.3	[0.2,0.5]	0.5	[0.4,0.6]
**Remoteness (ARIA+)**	Major city	392	4.8	1,523	31.1	26.3	Reference category		Reference category	
	Non major city	204	3.1	1,129	20.0	16.9	0.8	[0.5,1.3]	0.7	[0.6,0.9]
**Duration of pregnancy at first antenatal care visit**	Less than 20 weeks	519	4.6	2,198	26.4	21.8	Reference category		Reference category	
	20 + weeks	75	2.5	428	21.2	18.7	0.6	[0.5,0.8]	0.8	[0.7,0.9]
**TOTAL**		**596**	**4.0**	**2,652**	**25.2**	**21.2**	**N/A**			

## Discussion

This study examined demographic characteristics associated with smoking cessation in pregnancy and trends in these demographic characteristics between 2000 and 2011. Demographic characteristics associated with both higher rates of smoking in pregnancy and lower rates of smoking cessation during pregnancy included being a teenage mother, living outside major cities, being an Aboriginal person, and having an increasing number of previous pregnancies. These associations were observed in the first and last year of the study period. The study findings are consistent with previous findings from Australia [16,17] and internationally [18] that rates of smoking cessation during pregnancy are lower among teenage mothers, Aboriginal women, and women with more children.

This study indicates that smoking during pregnancy decreased steadily between 2000 and 2011, and smoking cessation during pregnancy was stable between 2000 and 2006 but increased markedly from 2007 onward. Many factors may have contributed to the changes in smoking behaviour observed in this study. At the population level, the adult smoking rate in NSW has been declining for over 30 years [25]. This decline is likely a result of sustained tobacco control strategies including taxation, advertising bans, mass media campaigns, and smoke-free environment legislation [26]. Recent initiatives include increases in the tobacco excise in 2010 and 2013, the introduction of bans on point-of-sale tobacco product displays in 2011, and the introduction of plain packaging of tobacco products in 2012 [26]. These population-level initiatives are likely to have raised general awareness of the risks of smoking among pregnant women, and contributed to the decline in smoking in pregnancy and increase in cessation during pregnancy observed in this study. However, the reason for the marked increase in rates of smoking cessation during pregnancy since 2007 is unclear. Although antenatal care providers are likely to provide some smoking cessation support as part of routine care, to the authors’ knowledge there have been no specific interventions targeting smoking in pregnancy, or changes in antenatal care practices since 2007, that would explain the sudden marked increase in cessation.

Despite the overall decline in smoking during pregnancy, demographic disparities in rates of smoking and smoking cessation in pregnancy remain substantial. Demographic groups including Aboriginal women, teenage mothers, women living outside major cities and women with three or more previous pregnancies remain significantly less likely to quit smoking during pregnancy. Moreover, although smoking cessation during pregnancy has increased in all demographic subgroups since 2000, the improvement in smoking cessation in these demographic groups is less than in other groups. To address these inequities, future efforts to reduce smoking in pregnancy may need to target specific population groups. Ongoing monitoring and evaluation is also valuable to assess the effectiveness of smoking cessation interventions in specific population groups, and the impact on pregnancy and birth outcomes.

Of particular concern are the high rates of smoking and low rates of smoking cessation among Aboriginal women. Aboriginal women continue to have the highest rates of smoking and lowest rates of smoking cessation during pregnancy of any demographic group, and the gap between Aboriginal and non-Aboriginal women is substantial. Reducing rates of smoking during pregnancy among Aboriginal women, and closing the gap in rates of smoking in pregnancy between Aboriginal and non-Aboriginal women, are key priorities in New South Wales [27]. The New South Wales Government state plan for the period 2011–2021 sets targets to reduce rates of smoking in pregnancy by 0.5% per year for non-Aboriginal women and by 2% per year for Aboriginal women. To achieve these targets, the New South Wales Ministry of Health is currently implementing *Quit for New Life*, a program facilitating improved provision of smoking cessation support to pregnant Aboriginal women. The impact of this program will be assessed and reported.

The observed associations between demographic characteristics and smoking cessation may in part reflect differential access to smoking cessation support. To the authors’ knowledge there are no Australian studies exploring factors associated with access to smoking cessation support during pregnancy, however overseas evidence suggests smoking cessation support is less likely to be provided to older women and poorer women [28]. In addition, there is evidence access to smoking cessation support varies by ethnicity, with one American study of pregnant women finding African American women were more likely, and Native American women less likely, to report receiving smoking cessation support compared to other women [29]. Several studies have also explored whether the effectiveness of smoking cessation support during pregnancy varies by demographic characteristics, however evidence is mixed. A recent systematic review concluded that psychosocial smoking cessation interventions during pregnancy are effective in reducing smoking in pregnancy. Sub-group analyses indicated psychosocial interventions have similar effectiveness among women from lower socioeconomic groups compared to other women, however trials in specific ethnic groups (African-American and Hispanic women) and Aboriginal women have found no treatment effect [30].

The main limitations of this study relate to the use of administrative data. First, the self-reported smoking status recorded in this dataset has not been validated. Self-reported data may underestimate the true rates of smoking in pregnancy as some women may choose not to disclose their smoking status, or unsuccessful attempts to stop smoking, to a health care provider. Second, the administrative dataset used for this analysis contains very limited information about smoking cessation. Using the available data, smoking cessation is defined as smoking during the first half of pregnancy but not in the second half. Smoking cessation which occurs during the second half of pregnancy is not recorded, as women who smoke at any time during the second half of pregnancy are recorded as smokers. Therefore it is likely that the Perinatal Data Collection underestimates rates of smoking cessation during late pregnancy. Further research is required to develop and validate robust measures of smoking cessation that could be incorporated into routine maternity data collection processes.

## Conclusions

Between 2000 and 2011, rates of smoking cessation during pregnancy improved dramatically across all demographic groups, particularly after 2007. However, specific demographic groups remain significantly less likely to quit smoking during pregnancy, including teenage mothers, women living outside major cities, Aboriginal women, and women with higher numbers of previous pregnancies. The findings suggest a need for targeted efforts to promote smoking cessation in specific population subgroups.
